# COVID-19 shed light on Virchow’s law of thrombosis

**DOI:** 10.4322/acr.2024.512

**Published:** 2024-08-28

**Authors:** Hubert Daisley, Oneka Acco, Martina Daisley, Dennecia George, Lilly Paul, Arlene Rampersad, Johann Daisley

**Affiliations:** 1 General Hospital, Department of Pathology, San Fernando, Trinidad and Tobago; 2 Scarborough General Hospital, Department of Pathology, Signal Hill, Trinidad and Tobago; 3 The University of the West Indies, Department of Pathology, Mona, Jamaica; 4 Princes Alexandra Hospital, Accident and Emergency Department The Valley, Anguilla

**Keywords:** Blood vessels, COVID-19, Thrombosis

## Abstract

Virchow’s law of thrombosis states that thrombosis in a vessel occurs as a combination of the following: (i) injury to the vessel wall, (ii) stasis of blood flow, and (iii) blood hypercoagulability. Injury to the wall includes infection/inflammation and/or injury to the resident cells of the wall. We postulate that in COVID-19, the SARS-CoV-2 virus directly infects the alveolar type II cell or directly or indirectly infects/injures the pericyte, promoting inflammation and interaction with endothelial cells, thereby causing a cascade of events leading to our observation that thrombosis occurred within the walls of the pulmonary vessels and not in the lumen of the vascular circulation.

## CENTRAL ISSUE

A thrombus is a blood clot in the lumen of blood vessels; it attaches to the site where it is formed and remains there, hindering the blood flow. Thrombosis of vessels has been governed by Virchow’s law of thrombosis.^1^

In Virchow’s triad of thrombosis, three factors are stated that predispose to vascular thrombosis, namely, (i) hypercoagulability of blood, (ii) alteration in blood flow in the vessels, and (iii) vessel wall injury/endothelial damage.^2^

In COVID-19, thrombosis of the pulmonary vessels was initiated within their walls,^3,4^ and not within their lumen, and was brought about by SARS CoV2 infection/injury of pericytes, causing inflammation, and interaction between dysfunctional pericyte, the vascular endothelial cell, and their biological micro-molecules in these locations. COVID-19 shed light on Virchow’s law of thrombosis, with thrombosis initiated within the pulmonary vessel wall and not within its lumen.

## DISCUSSION

**Figure 1:**
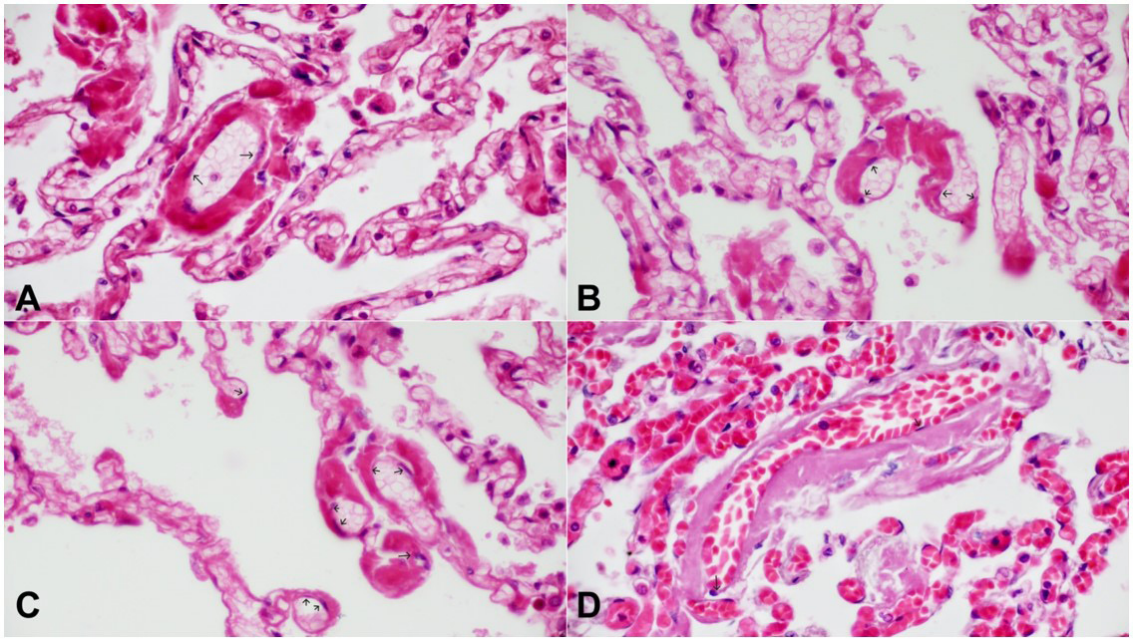
Photomicrographs of the lung. **A –** Alveolar capillary with thrombosis within the wall. Black arrow pointing to endothelial cells (H&E 10x40); **B –** Alveolar capillaries with thrombosis within the wall. Black arrow pointing to endothelial cells (H&E 10x40); **C** – Alveolar capillaries with thrombosis within the wall with various stages of lumen occlusion. Black arrow pointing to endothelial cells (H&E 10x40); **D –** Pulmonary venule with thrombosis within the wall. Black arrow pointing to endothelial cells (H&E 10x40).

We observed in our studies of Covid-19,^3,4^ that thrombosis of the alveolar capillaries began within the abluminal space where the pericytes reside. As these thrombi enlarge, they gradually narrow the lumens of the capillaries, eventually occluding them, as seen in Figures 1A, 1B, and 1C. Thrombi were also initiated within the walls of vessels in the other branches of the pulmonary microcirculation, such as venules and arterioles (Figure 1D).^5^

Within the abluminal spaces /walls of the alveolar capillaries and other vessels of the pulmonary microcirculation reside smooth muscle cells and other cells, such as the pericyte, which is prothrombotic. The pericyte is a multipotent cell that plays a role in maintaining the integrity of vascular endothelial cells. It has cytoplasmic finger-like projections that wrap around endothelial cells and form a barrier between endothelial cells and a shared basement membrane. It communicates with endothelial cells via peg sockets and gap junctions.^6^**^,^**^7^ It contributes to the alveolar basement membrane and contains numerous micro-molecules such as Tissue Factor within its cytoplasm/basement membrane, and together with von Willebrand factor and other factors from Weibel-Palade bodies in endothelial cells, and other factors in plasma, such as platelets, initiates inflammation and thrombosis.^8-12^

The pericyte with abundant ACE II receptor sites is the target cell for SARS-CoV-2, not the endothelial cell.^13-15^ The attachment of SARS-CoV-2 to alveolar pericytes or injury to pericytes caused by the attachment of SARS-CoV2 to alveolar type II cells causes dysfunction of pericytes and endothelial cells. Multiplication of the SARS-CoV-2 within the pericyte and or injury caused by cytokine release ultimately causes apoptosis of the pericyte,^11,16^ which causes dysfunction and disruption in the endothelial barrier, with leakage of plasma through peg junctions into the abluminal space. The leaked plasma, together with Tissue Factor and other micro-molecules from pericyte basement membrane/cytoplasm and factors from endothelial cells, produce an inflammatory response and a cascade of events, which leads to a hypercoagulable state, and the formation of fibrin thrombi within the walls of the pulmonary vessels.^17-21^

In COVID-19, the endothelial cells of pulmonary vessels are subjected to ischemic process/oxidative stress,^14,22,23^ which promotes endothelial dysfunction and additional plasma leakiness into the subendothelial space. Apoptotic or dysfunctional pericytes initiate inflammation, hypercoagulability, and thrombosis within the walls of the pulmonary vessels.

Andreeva et al.,^24^ found a continuous subendothelial network of pericyte-like cells in human vascular beds using 3G5 antibody, which recognizes o-acetylated disialoganglioside on the surface of capillary pericytes.

In large, medium, and small-size arteries, pericyte-like cells were identified in the inner intimal layer, predominantly in the sub-endothelium, the outer layer of the media, and in the adventitia in vasa vasora. In veins, pericytes were found in the sub-endothelial layer of the intima, in the media, and in the adventitia, where they were located in vasa vasora. These cells were revealed in the sub-endothelium of arterioles, venules, and capillaries of the pia mater encephali.^24:127^

These pericytes locations, which are all located in vessels walls, identified by Andreeva et al.,^24^ were the identical locations in the lungs where we observed thrombosis in COVID-19.^3,4^ Thrombosis began within the vessel’s walls, but not within their lumen. Juchem et al.,^25^ postulate that the exposure of sub-endothelial pericytes to blood components causes acute thrombotic events, further confirming the interaction of plasma and pericytes in thrombus formation in COVID-19.

Mazurek et al.,^26^ state that vascular diseases are characterized by perturbed interactions between different cell types, including endothelial cells, smooth muscle cells, pericytes, and macrophages. Such cell-cell interactions are poorly understood and likely integral to disease mechanisms. COVID-19 is a microvascular disease that, in part, is brought about by the interaction between pericytes, endothelial cells, and other vessel cells.

Billaud et al.,^27^ reported that adventitial vasa vasorum was the niche of perivascular progenitor cells, which included pericytes. It is within the sub-endothelial /abluminal spaces and within, and along the course of the adventitial vasa vasorum of the large, medium, and small pulmonary vessels, that thrombosis occurred in COVID-19.^4,14^

Thrombosis of the vasa vasorum, a small vessel the size of the alveolar capillary occurred in COVID-19, which played an integral role in pulmonary thromboembolism.^4^

In COVID-19, thrombosis did not originate within the lumen of either the alveolar-capillaries, or other vessels of the lung microcirculation or the lumen of the large, medium, or small pulmonary vessels. Still, thrombosis originated from within the walls of these vessels, which are the resident sites of the pericytes. This observation contradicts the definition of a thrombus, which conventionally is located within the lumen.

The vessel wall is not a mere structure providing a blood flow conduit. The vessel wall contains cells responsible for the vessel’s maintenance and integrity. Injury to the wall also implies injury to its resident cells and should be extended to Virchow’s law of thrombosis. In 1873, the French Scientist^28^ discovered the vessel mural cell, which was originally called Roguet cell, which was renamed some years later as the pericyte. It is now evident of the multipotent nature of the pericyte and its role in initiating inflammation.^13,29^

Injury to the vessel wall would, therefore, disrupt its cellular residents, which includes pericytes. Brotman et al.,^30^ revisited Virchow’s triad. They stated that Virchow’s contemporaries, Paget, Bochdaslek, and Cruveilhier, postulated then that thrombosis in the pulmonary artery occurred de novo and postulated that inflammation of the vessel wall was the primary impetus for thrombosis in the pulmonary artery. This is, in fact, the mechanism by which COVID-19 thrombosis occurs. Injury/SARS-CoV-2 infection/injury of pericytes or other neighboring cells such as the alveolar type ii cell in the pulmonary vessels wall mounts an inflammatory response,^14^ which is the genesis of pulmonary thrombosis in COVID-19.^31,32^

Hypercoagulable state, stasis of blood flow, and damage to the vessel wall is Virchow’s triad of thrombosis, which still prevails in the literature. Any one or a combination of the triad given together can bring about thrombosis in a vessel.

The pathophysiology supporting our observation that thrombosis occurred within the pulmonary vessel walls in COVID-19 is most complex and may involve other substrates stated in the discussion above.

Immunohistochemistry would be most useful in corroborate thrombosis in subendothelial locations since we used only the hematoxylin and eosin stains to observe the location of the endothelial cells and pericytes. ACE II receptor sites for SARS-CoV-2 spike protein are central to the pathogenesis of COVID-19 in the lung. Muhl et al.,^33^ in their study “The SARS-CoV-2 receptor ACE II is expressed in mouse pericytes but not endothelial cells: Implications for COVID-19 vascular research”^33:1089,^ used humanized mouse models and mouse-adapted SARS-CoV-2 virus in their study of COVID-19 pathogenesis. They found that ACE II expression occurred in pericytes in capillaries surrounding larger bronchi but not in alveoli capillaries or endothelial cells. However, ACE II receptor sites were strongly expressed in bronchial epithelial cells and alveolar type ii cells. The damage to the alveolar capillary endothelium may occur directly by the involvement of the pericyte or indirectly from the proximity of the endothelium to other infected cells such as the alveolar type ii cell. Whang et al.,^34^ proposed that cross-talk between alveolar epithelium and capillary endothelium mediates alveolar-capillary injury during SARS-CoV-2 infection.

Khan et al.,^13^ in their study “Preferential uptake of SARS-CoV-2 by pericytes potentiates vascular damage and permeability in an organoid model of the microvasculature”^13:3089^, hypothesized that within the vessel wall, “pericytes preferentially take up viral particles and mediate detachment, damage, and cell death, disrupting pericyte-endothelial cell cross-talk and increasing microvascular endothelial permeability, which promotes thrombotic and bleeding complications in the microcirculation”^13:3092^

The full understanding of the pulmonary pathophysiology in COVID-19 is yet to be elucidated, but injury to the pericyte and the endothelial cells play a central role in micro thrombosis. Further investigations need to be done to determine the role of the endothelial cell, the pericyte, the basement membrane, and other elements of the vessel wall, the alveolar cells, and plasma in the pulmonary pathophysiology of COVID-19. Research in this matter is ongoing.^35^

## CONCLUSION

In Covid-19

1. Injury/infection of pericyte/endothelial cells directly with SARS-CoV-2 or indirectly from SARS-CoV-2 attachment to alveolar type ii cells, promotes an inflammatory response.
2. The interaction of dysfunctional pericytes and, endothelial cells, and other agents brings about a hypercoagulable state with thrombosis occurring in the pulmonary vasculature in COVID-19.
3. We observed thrombosis within the pulmonary vessel walls but not in its lumen.

Hence, COVID-19 shed light on Virchow’s law of thrombosis.
